# Developing a Highly Stable *Carlina acaulis* Essential Oil Nanoemulsion for Managing *Lobesia botrana*

**DOI:** 10.3390/nano10091867

**Published:** 2020-09-18

**Authors:** Giovanni Benelli, Lucia Pavoni, Valeria Zeni, Renato Ricciardi, Francesca Cosci, Gloria Cacopardo, Saverio Gendusa, Eleonora Spinozzi, Riccardo Petrelli, Loredana Cappellacci, Filippo Maggi, Roman Pavela, Giulia Bonacucina, Andrea Lucchi

**Affiliations:** 1 Department of Agriculture, Food and Environment, University of Pisa, via del Borghetto 80, 56124 Pisa, Italy; valeriazeni93@gmail.com (V.Z.); renato_ricciardi@hotmail.it (R.R.); francesca.cosci1@virgilio.it (F.C.); gloria.cacopardo@hotmail.com (G.C.); andrea.lucchi@unipi.it (A.L.); 2 School of Pharmacy, University of Camerino, 62032 Camerino, Italy; lucia.pavoni@unicam.it (L.P.); saverio.gendusa@studenti.unicam.it (S.G.); eleonora.spinozzi@unicam.it (E.S.); riccardo.petrelli@unicam.it (R.P.); loredana.cappellacci@unicam.it (L.C.); filippo.maggi@unicam.it (F.M.); giulia.bonacucina@unicam.it (G.B.); 3 Crop Research Institute, Drnovska 507, 161 06 Prague, Czech Republic; pavela@vurv.cz; 4 Department of Plant Protection, Czech University of Life Sciences Prague, Kamycka 129, 165 00 Praha 6, Suchdol, Czech Republic

**Keywords:** European grapevine moth, green pesticide, insect pest, Integrated Pest Management, Larvicide, nano-insecticide, Tortricidae

## Abstract

The growing interest in the development of green pest management strategies is leading to the exploitation of essential oils (EOs) as promising botanical pesticides. In this respect, nanotechnology could efficiently support the use of EOs through their encapsulation into stable nanoformulations, such as nanoemulsions (NEs), to improve their stability and efficacy. This technology assures the improvement of the chemical stability, hydrophilicity, and environmental persistence of EOs, giving an added value for the fabrication of natural insecticides effective against a wide spectrum of insect vectors and pests of public and agronomical importance. *Carlina acaulis* (Asteraceae) root EO has been recently proposed as a promising ingredient of a new generation of botanical insecticides. In the present study, a highly stable *C. acaulis*-based NE was developed. Interestingly, such a nanosystem was able to encapsulate 6% (*w*/*w*) of *C. acaulis* EO, showing a mean diameter of around 140 nm and a SOR (surfactant-to-oil ratio) of 0.6. Its stability was evaluated in a storage period of six months and corroborated by an accelerated stability study. Therefore, the *C. acaulis* EO and *C. acaulis*-based NE were evaluated for their toxicity against 1st instar larvae of the European grapevine moth (EGVM), *Lobesia botrana* (Denis & Schiffermüller, 1775) (Lepidoptera: Tortricidae), a major vineyard pest. The chemical composition of *C. acaulis* EO was investigated by gas chromatography–mass spectrometry (GC–MS) revealing carlina oxide, a polyacetylene, as the main constituent. In toxicity assays, both the *C. acaulis* EO and the *C. acaulis*-based NE were highly toxic to *L*. *botrana* larvae, with LC_50_ values of 7.299 and 9.044 µL/mL for *C. acaulis* EO and NE, respectively. The *C. acaulis*-based NE represents a promising option to develop highly stable botanical insecticides for pest management. To date, this study represents the first evidence about the insecticidal toxicity of EOs and EO-based NEs against this major grapevine pest.

## 1. Introduction

The European grapevine moth (EGVM), *Lobesia botrana* (Denis & Schiffermüller, 1775) (Lepidoptera: Tortricidae), is a widespread and economically important pest of the grapevine worldwide. EGVM larvae feed on grape bunches (*Vitis vinifera* L.), reducing yield and increasing susceptibility to fungal and bacterial infections (i.e., botrytis and sour rot) [1].

Eco-friendly tools, including mating disruption and biopesticides (BPs) (mainly *Bacillus thuringiensis*), have been available against *L. botrana* for decades [2,3,4,5]. However, its control often requires the use of chemicals [6,7,8,9]. Finding valid and sustainable alternatives to insecticides is a key challenge for modern agriculture; side effects of insecticide use include environmental pollution, toxicity to non-target insects, and residues on food [10,11,12,13]. In this scenario, researchers are looking for new sustainable tools and products. Recently, they have focused on essential oils (EOs) as a new class of BPs to be employed in eco-friendly practices [14].

EOs are mixtures of plant metabolites, mainly monoterpenoids, sesquiterpenoids, and phenylpropanoids [14]; their insecticidal, acaricidal and nematocidal properties make them excellent alternatives to synthetic insecticides [14,15,16]. EOs are often characterized by two or three main compounds at high concentrations (20–85%) and other molecules at trace levels. A mechanism of action of EOs involves the inhibition of P450 cytochromes (i.e., these cytochromes are responsible for phase I metabolism of xenobiotics); other modes of actions include the neurotoxic activity-modulating octopaminergic system, gamma-aminobutyric acid (GABA) receptors and inhibiting acetylcholinesterase (AChE) [14].

EOs repellence [17,18], larvicidal [19,20,21], and insecticidal activities are proven on different arthropod pests of economic importance [22,23,24,25]. Among them, the EOs’ efficacy has also been investigated on some Lepidoptera. Good examples are represented by *Cydia pomonella* (Linneaus) (Lepidoptera: Tortricidae) [26], *Thaumetopoea pityocampa* (Denis & Schiffermüller) (Lepidoptera: Notodontidae) [27], *Cadra cautella* (Walker) (Lepidoptera: Pyralidae) [28] as well as *Spodoptera littoralis* (Boisd.) [29,30,31] and *Spodoptera litura* (Fabr.) (Lepidoptera: Noctuidae) [32,33,34]. However, current knowledge about EOs toxicity on *L. botrana* larvae is strictly limited [35].

Even though EOs represent promising BP ingredients, their use in Integrated Pest Management (IPM) programs is still scarce because their physico-chemical properties (i.e., poor water solubility, scarce stability, high volatility, thermal decomposition, and oxidative degradation) make them difficult to handle in field conditions. A solution to these difficulties is to coat or entrap EOs into a matrix. The encapsulation process enhances physico-chemical stability, prevents degradation of active agents, and improves the bioavailability of EOs [36,37]. Nanotechnology represents a suitable strategy to carry the EOs’ active principles, overcoming their physiochemical limitations; the small size of nano-systems increases active ingredients spreading, deposition, permeation, and provides controlled release on the target site. Among nano-delivery systems, nanoemulsions (NEs) represent an efficient, low-priced, and safe way to carry EOs [38].

As defined by Nikam et al. [39], NEs are kinetically stable “biphasic dispersions of two immiscible liquids: either water-in-oil (W/O) or oil-in-water (O/W) droplets stabilized by an amphiphilic surfactant”; in this way, protection from the surrounding environment, suitable spreading, and penetration of the bioactive molecules are guaranteed by the matrix and low surface and interfacial tension [40]. Toxicity of EO-based NEs was tested on several insects of agricultural and medical interest such as aphids [41,42,43], mosquitoes [44,45,46], stored-product beetles [47,48], and some Lepidoptera [49,50,51]. Furthermore, it was also highlighted that the bioactivity of EO-based NEs was often higher compared to the EOs themselves [52,53,54].

The insecticidal activity of EO-based NEs has never been evaluated against *L. botrana*. Herein, we decided to deepen our knowledge about EO and EO-based NE effectiveness against this harmful insect pest. For this purpose, we selected the EO obtained from the root of *Carlina acaulis* L. (Asteraceae), which has revealed to be promising as an active ingredient of botanical insecticides, highly effective against vectors and stored product insects [55,56].

*Carlina acaulis*, also called “piccolo cardo”, is a perennial herb growing on the mountainous soils of central Europe, up to 2000 m of altitude [57]. Being described in several official pharmacopoeias [58,59,60,61], this plant has been largely used in the European tradition as a remedy against several diseases [62,63,64]. Nowadays, its traditional use is still recognised in many European countries as a tonic, diuretic, anti-oedematous, anticancer, and antibiotic agent, and for the treatment of gastritis and cold [65,66,67,68]. Along with its various curative applications, *C. acaulis* is also described in the Italian list of botanicals to be used in food supplements [69] and in the BELFRIT (Belgium France Italy) list [70]. The EO obtained from the roots of this plant revealed as a major constituent (>90%) the polyacetylene 2–(3–phenylprop–1–yn–1–yl)–furan, also known as carlina oxide. The biological activities shown by this EO are noteworthy, but its innovative insecticidal potential is attracting the interest of the agrochemical industry.

In this scenario, reminding the importance of botanical EOs for the development of new sustainable pesticides, considering the promising insecticidal activities showed by the *C*. *acaulis* EO [56,71], and the limitations linked with its lipophilicity and volatility as well, herein a highly stable *C*. *acaulis*-based NE was developed. Furthermore, the *C*. *acaulis* EO and *C*. *acaulis*-based NE were evaluated for their toxicity against 1st instar larvae of *L*. *botrana*, a major grape pest worldwide.

## 2. Materials and Methods

### 2.1. Carlina acaulis Oil Extraction and Chemical Characterization

One kg of dry roots of *C. acaulis* obtained from A. Minardi and Figli S.r.l. (48012 Bagnacavallo RA, Italy), was firstly crushed using a shredder (Albrigi, mod. E0585, Stallavena, Verona, Italy), for then being put into a 10 L round flask with 6 L of distilled water. The roots were then subjected to hydrodistillation using a Clevenger-type apparatus for 8 h and using a heating system consisting of a Falc MA mantle (Falc Instruments, Treviglio, Italy). The EO, which showed a pale orangish colour, was obtained in a 0.4% yield (*w*/*w*). After the hydrodistillation process, the EO was decanted and separated from the aqueous layer, then dehydrated with an hydrous Na_2_SO_4_. Finally, it was collected in a vial closed with a polytetrafluoroethylene (PTFE)/silicone cap and kept at −20 °C until chemical analysis and subsequent biological assays. For the chemical characterization of the *C. acaulis* EO, the analysis was conducted using an Agilent 6890N gas chromatograph furnished of a single quadrupole 5973N mass spectrometer and an auto-sampler 7863 (Agilent, Wilmingotn, DE). The column used for the separation was an HP-5 MS capillary column (30 m length, 0.25 mm i.d., 0.1 μm film thickness; 5% phenylmethylpolysiloxane), supplied by Agilent (Folsom, CA, USA). The column was allowed to reach initially a temperature of 60 °C for 5 min, then it was raised up to 200 °C at 4 °C/min and finally to 280 °C at 11 °C/min for 15 min. The temperature of the injector and detector was set at 280 °C. The mobile phase used was constituted of 99.9% of He, with a flow of 1 mL/min. Before injection, the EO was diluted 1:100 in *n*-hexane, and then 1 µL was injected in split mode (1:50). The peak acquisition was achieved with electron impact (EI, 70 eV) mode in the range 29–400 *m/z*. The chromatograms obtained were analysed using the MSD ChemStation software (Agilent, Version G1701DA D.01.00) and the NIST Mass Spectral Search Program for the NIST/EPA/NIH EI and NIST Tandem Mass Spectral Library v. 2.3. The retention index (RI) was calculated using a mix of *n*–alkanes (C_7_–C_30_, Sigma-Aldrich, Milan, Italy), using the Vanden Dool and Kratz formula [72].

### 2.2. Preparation and Characterization of Carlina acaulis Essential Oil (EO) Nanoemulsion

*Carlina acaulis* EO-based NE was obtained through a high-energy method by using a high-pressure homogenizer. It was prepared according to the procedure reported by Rosi Cappellani et al. [73]. Briefly, 6% (*w*/*w*) of *C. acaulis* EO was added dropwise to a 4% (*w*/*w*) of surfactant (Polysorbate 80, Sigma-Aldrich) aqueous solution under high-speed stirring (Ultraturrax T25 basic, IKA^®^ Werke GmbH and Co.KG, Staufen, Germany) for 5 min at 9500 rpm. The obtained emulsion was then subjected to homogenization by means of a French Pressure Cell Press (American Instrument Company, AMINCO, Silver Spring, MD, USA) for four cycles at the pressure of 130 MPa.

Visual characterization of NE was performed by a polarizing optical microscope (MT9000, Meiji Techno Co. Ltd., Saitama, Japan) equipped with a 3-megapixel complementary metal oxide semiconductor (CMOS) sensor camera (Invenio 3S, DeltaPix, Smorum, Denmark).

Particle size measurements were carried out through dynamic light scattering (DLS) analyses by using a Zetasizer nanoS (Malvern Instrument, Malvern, UK) equipped with a backscattered light detector working at 173°. One mL of the sample was inserted into a disposable cuvette and analysed at 25 °C, following a temperature equilibration time (180 s).

### 2.3. Nanoemulsion Stability Studies

#### 2.3.1. Long-Term Stability

The sample was stored at room temperature and 12:12 (L:D) h for up to six months. The physico-chemical stability of the samples was evaluated by repeating DLS analysis at different time points: 0 day (t0), 1 month (t1), 3 months (t3), and 6 months (t6).

#### 2.3.2. Accelerated Stability Test

The thermodynamic stability of *C. acaulis* EO NE was evaluated through a three phases (centrifugation, heating/cooling cycles, and freeze/thaw cycles) test, according to the protocol reported by Alkilani et al. [74] with some modifications.

1. Centrifugation: the sample was centrifuged at 9000 G for 30 min. If it did not show any phase separation, the heating-cooling cycle was performed.
2. Heating-cooling cycle: the sample underwent three cycles from refrigerator temperature (4 °C) to 40 °C, with a storage period at each temperature of 48 h. If stable at these temperatures, the freeze-thaw cycle was performed.
3. Freeze-thaw cycle: three freeze-thaw cycles between −21 °C and +25 °C were performed, with a storage time at each temperature of 48 h.

At the end of each phase, the sample was evaluated through visual inspection and DLS analysis.

### 2.4. Lobesia botrana Mass-Rearing

*Lobesia botrana* young instars tested in our bioassays were from a laboratory mass-rearing kept at the Entomology lab, University of Pisa. Adults were reared inside a plastic bottle and fed with a liquid diet. Eggs were collected every 2 days and placed into a plastic tray, previously drilled to allow airflow; each tray contained a piece of artificial food medium. Semi-synthetic larval diet is based on Gabel et al. [75] recipe (for 1 kg: deionized water 750 mL, agar-agar 15 g, sucrose 30 g, alfalfa flour 25 g, brewer’s yeast 18 g, salts of Wessen 12.5 g, cholesterol 1.25 g, wheat germ 90 g, casein 40 g, sorbic acid 2 g, ascorbic acid 10 g, vitamins wanderzahnt 7.5 g, tetracycline 1.25 g, propionic acid 2.5 g, linoleic acid 1 mL, sunflower oil 2 mL); emerged adults were transferred into a new polyvinyl chloride (PVC) bottle. The rearing was maintained at a temperature of 25 ± 1 °C, R.H. 70 ± 10% and 16:8 (L:D) photoperiod.

### 2.5. Insecticidal Activity on Lobesia botrana 

The insecticidal activity of EO and NE of *C. acaulis* on *L. botrana* was tested adapting the method by Bosch et al. [76] originally developed for insecticide toxicity assessment on *C. pomonella*. A 32 μL-drop of NE or EO formulation was deposited on the surface of a piece of semi-synthetic diet (4 × 4 × 1 cm) using a micropipette. The solution was evenly distributed using a humidified brush and allowed to dry for 2 h. Sixteen 1st instar larvae (L1) of *L. botrana* were deposited on each piece of diet and individualized within a gelatine capsule (00, Fagron, Quarto Inferiore, Bologna, Italy). Each piece of the diet with the larvae was placed in a closed plastic box to avoid desiccation.

Larval mortality was observed 96 h later, gelatine capsules were removed, and the diet was observed under a binocular microscope for larvae inside the diet. A larva was considered dead if it did not respond to a gentle touch with a small brush. Missing larvae were considered escaped and subtracted from the number of treated larvae. Seven concentrations of *C. acaulis* EO (1, 2.5, 6, 7.5, 8, 10, 30 µL/mL) and six concentrations of *C. acaulis* NE (5, 7.5, 8, 10, 30, 60 µL/mL) were tested, water was used as solvent to prepare the dilutions.

To validate the method described above, we also tested positive and negative controls. The positive control was a commercial insecticide, Spinosad (Laser^®^, Dow) tested at the tab dose (15 mL/hL); the negative control was 0.17% Polysorbate 80 + 99.83% of H_2_O for NE and H_2_O + dimethyl sulfoxide (DMSO) at the same concentration of the EO. At least three replicas for each concentration of EO, NE, positive and negative control were performed. For each tested product concentration, four duplicate trials were carried out; replicates were conducted over different days to account for any daily variability. Each concentration was always replicated with a new concentration series prepared for each replicate. All experiments were performed at laboratory conditions of 22 ± 1 °C, R.H. 45 ± 5%, and photoperiod 16:8 (L:D).

### 2.6. Statistical Analysis

*Lobesia botrana* mortality (%) was arcsine√ transformed before performing an analysis of variance (ANOVA, two factors as fixed effects) followed by Tukey’s honestly significant difference (HSD) test (*p* < 0.05). The experimental mortality was corrected with Abbott’s formula, if control mortality ranged from 1 to 20%; if control mortality was > 20% experiments were discarded and repeated [77]. LC_10_, LC_30_, LC_50,_ and LC_90_ with associated 95% confidence interval (CI) and chi-squares, were estimated using probit analysis [78]. JMP 9 (SAS) software was used for all analyses, and *p* = 0.05 was selected as a threshold to assess significant differences.

## 3. Results and Discussion

### 3.1. Essential Oil Chemical Composition

**Figure 1:**
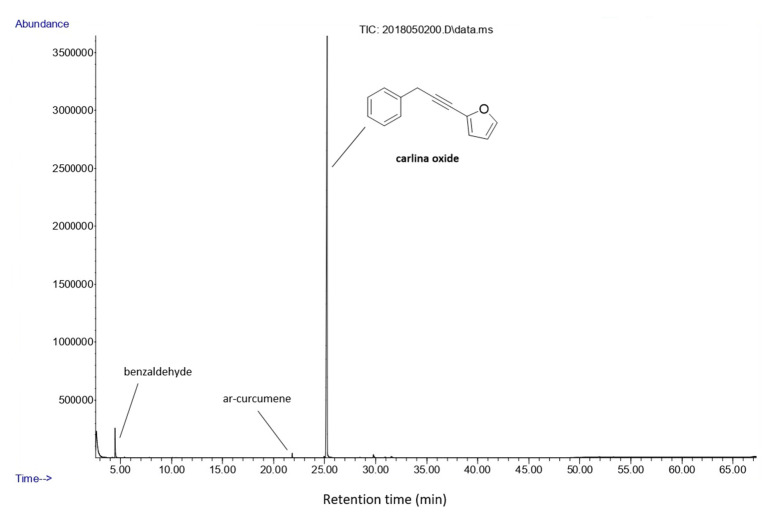
Gas chromatography–mass spectrometry (GC–MS) chromatogram of the essential oil obtained from the roots of *Carlina acaulis*. The separation of peaks was achieved using a HP-5MS (5% phenylmethylpolysiloxane, 30 m length × 0.25 mm internal diameter, 0.1 μm film thickness).

Through gas chromatography–mass spectrometry (GC–MS) analysis, the EO extracted from the roots of *C*. *acaulis* was characterised and the data obtained were in accordance with the work of Benelli et al. [56]. Seven compounds were identified, among which carlina oxide was the predominant EO component, comprising 94.6% of the relative content. Other compounds were identified, such as the aromatic benzaldehyde (3.1%) and the sesquiterpene *ar*-curcumene (0.4%) (Figure 1). Acetophenone, benzyl methyl ketone, camphor, and carvone were detected at trace levels. 

### 3.2. Preparation and Characterization of the Essential Oil Nanoemulsion

NEs are colloidal systems offering a great advantage to encapsulate a higher amount of oil phase respective to similar nanosystems, i.e., microemulsions [79]. In fact, such a system has allowed to vehiculate 6% (*w*/*w*) of EO, respective to at least 1.5% (*w*/*w*) encapsulated into microemulsions, as reported in previous studies [80,81]. Moreover, NEs require a meager amount of surfactant (4% *w*/*w*), with a surfactant-to-oil ratio (SOR) of around 0.6, respective to that of microemulsions, that is generally higher than 2 (SOR > 2) [82].

However, NEs are energetically disadvantaged nanosystems because they have a higher free energy level respective to that of the two separated phases (water + oil). Thus, to produce a colloidal system, an external energetic input is required to overcome the activation energy barrier separating the two phases. In this respect, one of the most commonly used methods is the homogenization process. It is a high-energy method that consists of a 2-step procedure [83]. The first step gives rise to an emulsion, characterized by oil droplets mainly in the micrometric range, through the high-speed stirring process of the oil and water phases [82]. The second step, the high-pressure homogenization, provides the breakage of oil droplets into small ones by forcing the material to flow through small nozzles or valves by exerting very high pressures with a piston pump. During the flow, the emulsion is exposed to shear stress able to give rise to nanometric oily droplets [84].

**Figure 2:**
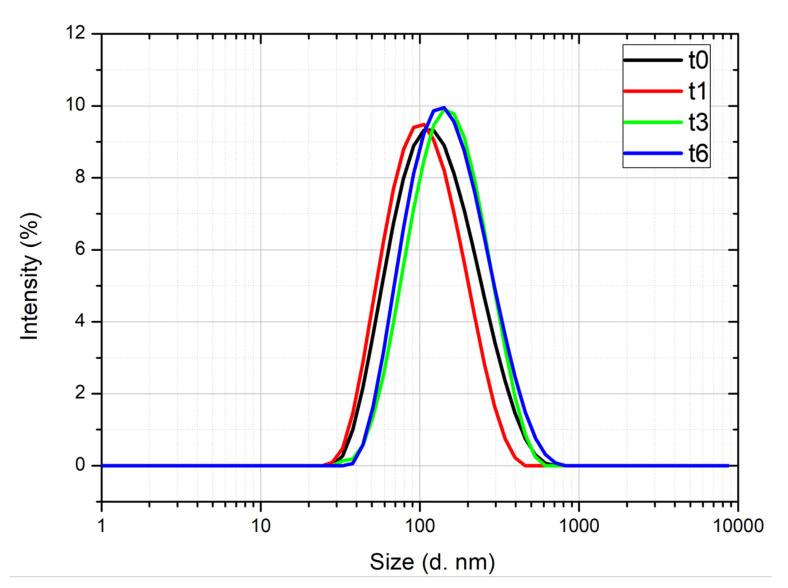
Dynamic light scattering (DLS) traces of *Carlina acaulis* essential oil-based nanoemulsion, at different time points: 0 day (t0), 1 month (t1), 3 months (t3), 6 months (t6).

For the achievement of *C. acaulis* EO-based NE, the sample was subjected to a pressure of 130 MPa four times. The sample showed a monomodal size distribution with a size in the nanometric range. In particular, the droplets’ population had a mean diameter centred around 140 nm (Figure 2, black line). DLS analysis recorded Z-average and PDI (polydispersity index) values of 98.85 and 0.33, respectively. The Z-average value or Z-average mean used in DLS is a parameter, also known as the cumulants mean, that can be defined as the “harmonic intensity averaged particle diameter”. Assuming that the particle population is a simple Gaussian distribution, the Z-average is the mean, and the PDI is related to the width of this simple distribution. Thus, the smaller the PDI (≤ 0.3), the more monodispersed the system will be [85].

**Figure 3:**
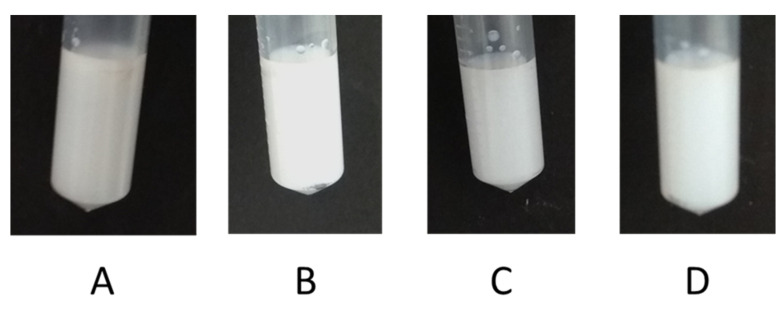
*Carlina acaulis* essential oil nanoemulsion (EO NE) at t0 (**A**), after the centrifugation (**B**), after the heating–cooling cycles (**C**) and after the freeze-thaw cycles (**D**).

The *C. acaulis* EO NE showed optimal stability at room temperature, evaluated for a storage period of six months. As reported in Figure 2, the size of the oil droplets remained almost unchanged, with a slight shift of mean hydrodynamic diameter from 143.9 nm at t0 to 170.2 nm after 6 months. These results proved the thermodynamic stability of the system. It was also confirmed by the accelerated stability test, generally used to predict the thermodynamic stability of the system for long-term periods. The accelerated stability was evaluated via centrifugation, heating–cooling cycles, and finally, freeze-thaw cycles stress tests. The NE showed a good physical stability at the centrifugal forces (Figure 3B) and remained almost unaltered to the heat–cool cycles. No signs of creaming, phase separation or cracking were detected (Figure 3C). These images were also corroborated by DLS analysis results (Table 1), that revealed the conservation of the internal phase structure, being the Z-average and PDI values almost unchanged with respect to those of the NE at t0. A slight creaming effect was observed when the NE was frozen at the temperature of −21 °C. However, its homogeneity was recovered upon the thawing phase (Figure 3D). A similar result was reported by Ammar et al. [86], who attributed this transient instability to the low temperature leading to the coagulation of the internal phase. This perturbation of the systems was revealed by the increased value of the Z-average after the freeze-thaw cycles, as reported in Table 1. However, the size of the oil phase was kept below 200 nm, which is the upper limit generally established by authors for NEs [87].

Therefore, given the results achieved by the stability study, this *C. acaulis* EO-based NE can be considered a physico-chemically stable nanosystem.

### 3.3. Insecticidal Activity on Lobesia botrana

Larval mortality in exposed *L*. *botrana* individuals was directly proportional to *C*. *acaulis* EO and *C*. *acaulis*-based NE concentrations (*F_6,23_* = 40.47, *p* < 0.0001; *F_5,23_* = 27.22, *p* < 0.0001, respectively); significant larvicidal activity was observed starting from 2.5 µL/mL of EO and 8.0 µL/mL of NE. Comparable concentrations of *C*. *acaulis* EO showed higher larvicidal activity over the *C*. *acaulis* EO NE. As reported in Table 2, 50% of larval mortality was achieved testing a concentration equal to 7.29 ± 0.25 µL/mL of *C*. *acaulis* EO and 9.04 ± 0.39 µL/mL of *C*. *acaulis* NE. Besides, the LC_90_ of *C*. *acaulis* EO was lower than that of *C*. *acaulis*-based NE (10.92 ± 1.40 µL/mL and 17.70 ± 4.48 µL/mL, respectively); 100% larval mortality was achieved with the positive control represented by a semi-synthetic diet treated with the positive control spinosad (Laser^®^) at the label dose (i.e., 150 ppm).

It is difficult to compare our results with the findings by other authors as, to the best of our knowledge, research on the insecticidal efficacy of plant EOs against *L*. *botrana* is extremely limited. Only one study was retrieved, where Avgin et al. [35] tested 5 essential oils from seeds or aerial parts of aromatic plants such as *Thymus vulgaris* L., *Mentha* x *piperita* L., *Foeniculum vulgare* Mill., *Rosmarinus officinalis* L. and *Carum carvi* L. on field-collected grapes. The authors found that the EO from *C. carvi* was the most effective, since at a concentration of 25 µL on 20 g of grapes it achieved >96% mortality on *L. botrana* larvae. Most research about the EO efficacy on *L. botrana* was undertaken to explore any changes in adults’ behaviour in response to EO aroma [88], aimed at using EOs to improve pest control strategies [89]. As far as we know, our study is the first that assesses EO efficacy on the mortality of freshly hatched *L. botrana* larvae as the usual target of insecticide application. Also, the study of EO-based NE efficacy on the larvae of phytophagous lepidopteran species is only beginning, and few papers on NE efficacy on moth pests exist so far [49,50,51], although EOs have been known to provide very good insecticidal effects on pests including phytophagous moth larvae [26,27]. Moreover, as indicated by previous studies, EO-based NEs actually show very promising effects, often significantly higher if compared to EOs [49,52,53].

*Carlina acaulis* EO was obtained from roots of carline thistle, and its main component is carlina oxide (~94%), one of the oldest known polyacetylenes. A recent study conducted by Benelli et al. [56] proved carlina oxide as a mild acetylcholinesterase (AChE) inhibitor. It has also been documented that polyacetylenes cause phototoxicity in insects [90], and are able to modulate GABA_A_ receptors [91]. Recently, the effectiveness of *C. acaulis* EO has been demonstrated on other insect species, showing acute and sub-lethal toxicity on highly important pests and vectors, such as the southern house mosquito, *Culex quinquefasciatus* (Say) (Diptera: Culicidae) (LC_50_ = 1.31 μg mL^−1^) [56] and the common housefly, *Musca domestica* (L.) (Diptera: Muscidae) (LC_50_ = 2.74 (♂) and 5.96 (♀) μg fly^−1^) [71]. Moreover, simulating a small-scale maize conservation environment, the *C. acaulis* EO led to relevant insecticidal activity against *Prostephanus truncatus* (Horn) (Coleoptera: Bostrychidae), with 500 ppm killing >97% of adult beetles within three days [55]. Either the effectiveness, as well as the availability and low costs of the *C. acaulis* EO, encourage further experimentation on EGVM for green pesticide development.

The comparison of the LC values obtained by testing *C. acaulis* EO and the corresponding NE, showed a comparable larvicidal activity. From the values reported in Table 2, it is possible to observe a higher insecticidal activity of the pure EO (LC_90_ = 10.922 ± 1.40 µL/mL) over the NE (LC_90_ = 17.706 ± 4.48 µL/mL). On the other hand, the NE contains 6% of EO, a value 16 times lower respect to pure EO used as reference. This shows that the EO encapsulated in the NE is more active than the pure *C. acaulis* EO, if considered at the same concentration. The increase in the larvicidal activity of pure EO encapsulated into the NE could be attributed to a better interaction between the active substance and the target site. First, the NE, providing a greater dispersion of the lipophilic substance (EO) in the aqueous phase, allows the diffusion of the EO in the *L. botrana* growth environment. Furthermore, the NE is able to increase the concentration of the EO at the interface, leading to a better and direct interaction with the biological components of the target. Moreover, the small size and large surface area of the NE-encapsulated EO droplets allow an increased absorption and cellular penetration into the target site. Therefore, the encapsulated EO can exert its larvicidal activity even at lower concentrations than pure EO. Finally, the NE appears promising in controlling the growth of *L. botrana*, not only for the larvicidal potential but also for the improvement of the physico-chemical properties and stability of the EO [38].

The efficacy of EOs including EO-based NEs may not be necessarily related only to acute or chronic toxicity, but, as already shown, even sub-lethal EO doses or concentrations may reduce the vitality, fertility, and longevity of insects [92,93] including harmful moths [26,27,28]. This phenomenon was also confirmed for the EO from *C. acaulis*, although for other insect species [56,71] and, therefore, any potential effect of sub-lethal concentrations should be studied for *L. botrana* as well. Similarly, the possibility of enhancing the insecticidal activity of the EO from *C. acaulis* using a suitable synergistic mixture with other EOs or their major constituents should be considered in further studies.

This study opens a new perspective on *L. botrana* management using botanical pesticides. It highlights the potential of Asteraceae EOs as valid alternatives to chemical insecticides, because of their low human toxicity, rapid degradation, low environmental impact, and reduced likelihood to trigger insecticide resistance [14,94,95]. The lack of physico-chemical stability makes EOs difficult to handle in open field conditions. However, the adoption of nano-delivery systems (e.g., NEs) increases EOs stability and solubility, improving their delivery, and establishing a sustained release of the active ingredients [38]. The adoption of nanotechnology in IPM showed it to be useful to overcome EOs’ drawbacks and to amend their efficacy as biopesticides.

Further studies should be conducted on the larvicidal and adulticidal activity of EOs and EO-based NEs on *L. botrana* to find a valid substance to test in the open field. Moreover, as highlighted by Pavoni et al. [79], it is crucial to consider that a lot of EO-based NEs contain several, non eco-friendly ingredients (i.e., polysorbates). Thus, further research is needed to evaluate the effects of nano-encapsulation on EO toxicological profiles.

As mentioned above, the use of EOs to eliminate insects is an alternative pest control method that minimises any harmful effects on the environment. Since EOs are chemicals commonly found in nature, being contained in almost all vascular plants, and have been shown to be very friendly to non-target organisms, botanical insecticides based on EOs can be considered relatively safe for the environment [14,38,94]. Moreover, as EOs are highly volatile, only minimal problems with their residues are expected when used in soil and aquatic ecosystems [94]. We are aware that further studies on the effects of *C. acaulis* EO on non-target organisms will be needed to confirm environmental safety for this EO. Although solvents are usually added to EO-based formulations [79], NEs used in this study contain no solvents and are based on a surfactant with no effects in terms of eco-toxicity given its high level of biodegradability.

## 4. Conclusions

The present study highlighted the promising potential of the *C*. *acaulis* root EO as an effective ingredient for botanical insecticide development. This EO showed high insecticidal efficacy against 1st instar larvae of *L. botrana*, a major pest affecting grape cultivation, causing yearly significant economic damages. Moreover, this research supported the real-world applications of the *C. acaulis* EO through its encapsulation into a nanoformulation. The EO-based NE guarantees the conservation of the insecticidal activity while ensuring dispersibility in the environment as well as its stability along time. Although the results encourage the use of *C. acaulis* EO in the agricultural field, especially in organic farming, further investigations are needed to evaluate its eco-toxicological profile. Similarly, further studies are needed to reveal the effects of lethal and sub-lethal concentrations on fertility, longevity, and behaviour of *L. botrana*.

## Figures and Tables

**Table 1:** Thermodynamic stability evaluation, in terms of Z-average, polydispersity index (PDI), creaming, and phase separation, of the *Carlina acaulis* essential oil (EO) nanoemulsion through the accelerated stability test.

	Z-Average *	SD	PDI *	SD	Creaming	Phase Separation
t0	98.85	1.41	0.33	0.04	-	-
Post CENTRIFUGATION	95.54	1.32	0.31	0.031	NO	NO
Post HEATING-COOLING	90.68	1.32	0.33	0.02	NO	NO
Post FREEZE-THAW	153.93	1.58	0.28	0.005	NO **	NO

* The value is the mean of three measurements. ** The creaming phenomenon was observed only after the freezing of the sample. However, at the end of the cycles, after the thawing process, the sample did not more show creaming.

**Table 2:** Larvicidal activity of *Carlina acaulis* essential oil (EO) and its 6% nanoemulsion (NE) against 1st instar larvae of *Lobesia botrana*.

Tested Product	LC_10_^1^ ± SE ^2^ (CI_95_) ^3^ (µL/mL)	LC_30_ ± SE (CI_95_) (µL/mL)	LC_50_ ± SE (CI_95_) (µL/mL)	LC_90_ ± SE (CI_95_) (µL/mL)	*χ* ^2^	*p-*value
*C. acaulis* EO	4.87 ± 0.49 (3.9–5.4)	6.19 ± 0.31 (5.6–6.5)	7.29 ± 0.25 (6.9–7.6)	10.92 ± 1.40 (9.7–13.6)	1.158	0.563 n.s.^4^
*C. acaulis* EO in NE	6.24 ± 0.58 (5.1–6.8)	7.77 ± 0.33 (7.2–8.1)	9.04 ± 0.39 (8.6–9.7)	17.70 ± 4.48 (15.4–27.5)	1.257	0.262 n.s.

^1^ LC = lethal concentration killing 10%(LC_10_), 30% (LC_30_), 50%(LC_50_) or 90% (LC_90_) of the exposed population; ^2^ SE = standard error; ^3^ CI_95_ = 95% confidence interval; ^4^ n.s. = not significant (*p* > 0.05). Positive control spinosad (Laser^®^) tested at tab dose (150 ppm) achieved 100% mortality.
